# Supplementary material for: A Critical View of the Application of the APEX Software (Aqueous Photochemistry of Environmentally-Occurring Xenobiotics) to Predict Photoreaction Kinetics in Surface Freshwaters
Source: Molecules. 2019 Dec 18;25(1):9. doi: 10.3390/molecules25010009 (PMC7017383; doi:10.3390/molecules25010009)
Supplement: Supplementary file 1 [file molecules-25-00009-s001.zip › 3.2.4_gcc-4.4.0.zip!/3.2.4_gcc-4.4.0/3.2.4_gcc-4.4.0/doc/octave/html/interpreter/Fonts.html]

Fonts - Untitled


Next: Evaluation Notation,
Up: Conventions


---

#### 1.3.1 Fonts

Examples of Octave code appear in this font or form: `svd (a)`.
Names that represent variables or function arguments appear
in this font or form: first-number. Commands that you type at the
shell prompt appear in this font or form: octave --no-init-file.
Commands that you type at the Octave prompt sometimes appear in this font
or form: `foo --bar --baz`. Specific keys on your keyboard appear
in this font or form: <ANY>.
